# Fly Ash Waste Recycling by Pt/TiO_2_ Incorporation for Industrial Dye Removal

**DOI:** 10.3390/ijerph18083887

**Published:** 2021-04-07

**Authors:** Maria Visa, Mihaela Cosnita, Macedon Moldovan, Cosmina Andreea Marin, Maria Mihaly

**Affiliations:** 1 Research Center: Renewable Energy Systems and Recycling, Transilvania University of Brasov, Eroilor 29, 500036 Brasov, Romania; maria.visa@unitbv.ro (M.V.); mihaela.cosnita@unitbv.ro (M.C.); macedon.moldovan@unitbv.ro (M.M.); 2 Research Center for Environmental Protection and Eco-Friendly Technologies (CPMTE), University POLITEHNICA of Bucharest, 060042 București, Romania; lazarcosmina@ymail.com; 3 Inorganic Chemistry, Physical Chemistry and Electrochemistry Department, Faculty of Applied Chemistry and Materials Science, University POLITEHNICA of Bucharest, 060042 București, Romania

**Keywords:** fly ash waste, platinum nanoparticles, industrial dyes, adsorption, photodegradation

## Abstract

New materials are obtained by transforming fly ash wastes into a valuable composite, with tandem adsorption and photodegradation properties. Mild hydrothermal synthesis, from titanium dioxide, platinum nanoparticles and zeolite materials obtained from a waste, fly ash, as support, was involved in the composite preparation. The platinum nanoparticles extended the photocatalytic activity of the composite in visible range (Eg = 2.1 eV). The efficiency of tandem adsorption and photocatalytic activity of the new composite were determined to be 25% for Bemacid Blau and 43.89% for Bemacid Rot after 360 min, the irradiation time. The addition of H_2_O_2_ improves the process efficiency up to 80.70% and 93.43%, respectively. The Pt nanoparticle (PtNP) contribution led to the band gap energy change to Vis light (400 nm), thus suggesting the possibility of photocatalysis under the action of a fraction of natural light.

## 1. Introduction

Increasingly intense and diverse industrial activity raises major issues of environmental protection. It is, on the one hand, the discharge of pollutants into the environment, either accidentally or due to the inefficiency of removal methods. For example, dyes are dangerous pollutants and must be removed immediately [1]. Most of these are carcinogenic, harmful and reduce the light penetration in aqueous systems causing the photosynthesis process, making them harmful to aquatic live and of course to human health [2,3].

On the other hand, there is the issue of industrial waste dumps. Among them are the fly ash (FA) from power plants. Depending upon the source and composition of the coal being burned, the components of fly ash vary considerably, but all fly ash includes substantial amounts of silicon dioxide (SiO_2_) (both amorphous and crystalline), aluminum oxide (Al_2_O_3_), iron oxide (Fe_2_O_3_) and calcium oxide (CaO) [4]. These physical, chemical and mineralogical properties suggest the opportunities for use and disposal.

A contextual approach, namely, the use of fly ash waste to develop effective solutions in the removal of industrial dyes, can help to reduce their negative effect on the environment.

The industrial dyes have in their chemical structure one, two or more aromatic rings with chromophore groups, such as the azo group (–N=N–), which gives them high stability, and they are therefore difficult to degrade [5,6]. Several processes have been investigated: coagulation [7], adsorption [5], aerobic or anaerobic biosorption [8,9] and advanced chemical oxidation or photochemical degradation [10,11,12,13]. Of these, photochemical degradation is one of the most effective.

Photochemical degradation uses different semiconductor oxides (TiO_2_, WO_3_, SnO_2_, ZnO, combined oxides [14,15], modified oxides [16]) with photocatalytic activity. Titanium dioxide (TiO_2_) is the most widely used photocatalyst because of its properties, such as non-toxicity, low cost, strong oxidation power and high chemical stability. One of the shortcomings of this procedure is the difficulty to separate the liquid and the solid photocatalyst after removing the pollutants. For this reason, researchers have explored many solid substrates [17,18,19] to immobilize WO_3_ and TiO_2_ [20]. Thus, FA, given its composition (especially SiO_2_, Al_2_O_3_ and unburned carbon), could be a suitable support material to be optimized as an adsorbent with potential photocatalytic properties depending on the burned coal source and composition, as small quantities of TiO_2_, MnO_2_ can be found in FA. The limited efficiency of photocatalytic activity comes from the high rate of electron-hole recombination into semiconductor particles, TiO_2_, in this case. Various heterogeneous catalysts have been extensively studied to enhance the efficiency of organic pollutant degradation, such as doping metal ions into the TiO_2_ lattice [21], noble metal (Pt, Pd, Au, Ag) addition to TiO_2_ surface [22,23,24,25,26], depositing on an inert matrix such as fly ash [27] or incorporation of photoactive TiO_2_ into an aluminosilicate inorganic polymer [28].

Based on these considerations, an excellent research opportunity is created, and the main objective of this study is to use fly ash waste as a source for photoabsorbent material production. This paper aims to demonstrate, through a detailed investigation, a technically feasible way to combine non-energy raw materials from different sources (Romanian fly ash resulting from the burning of harder, older anthracite and bituminous coal, in this case) and quality (F type—the sum of SiO_2_, Al_2_O_3_ and Fe_2_O_3_ is over 70%) with TiO_2_ semiconductor and Pt nanoparticles (PtNPs). The influence of H_2_O_2_ on the degradation efficiency of industrial dyes is also evaluated.

Finally, a hybrid procedure, based on adsorption, photo oxidation and chemical oxidation, is proposed to efficiently degrade some industrial dyes, Bemacid Rot (BR) and Bemacid Blau (BB).

## 2. Materials and Methods

### 2.1. Materials

Fly ash (FA) used in this study was collected from Heating Plant CET Brasov, Romania. This FA is a cheap material, a poly-oxide compound with a high percentage of SiO_2_ (53.32%), Al_2_O_3_ (22.05%) and of Fe_2_O_3_ (8.97%). According to the American Society for Testing and Materials (ASTM) standard C-618-2a [29], this fly ash is of F type (the sum of SiO_2_, Al_2_O_3_ and Fe_2_O_3_ is over 70%). In addition to these major oxides, others were identified (TiO_2_—1.07%, Mn_2_O_3_—0.08%) in the FA composition that act as photocatalysts for organic pollutant photodegradation. The unburned carbon (1.58%) can act as an efficient adsorbent of dyes and heavy metals.

The TiO_2_ content was increased by FA mixing with Degussa P25 (anatase/rutile ratio = 3:1), with 55 m^2^/g BET (Brunauer-Emmett-Teller) surface, particle size of around 30 nm and 97% purity.

Stable dispersion of cysteamine modified platinum nanoparticles (Pt-Cys NPs) was achieved using polyoxyethylene-4-lauryl ether (Brij 30), n-heptane and cysteamine monoclorhidrate (CS) provided by Sigma-Aldrich. Ultrapure Millipore water was used for the preparation of the samples. All the other reagents were analytical grade and were used without further purification.

**Figure 1:**
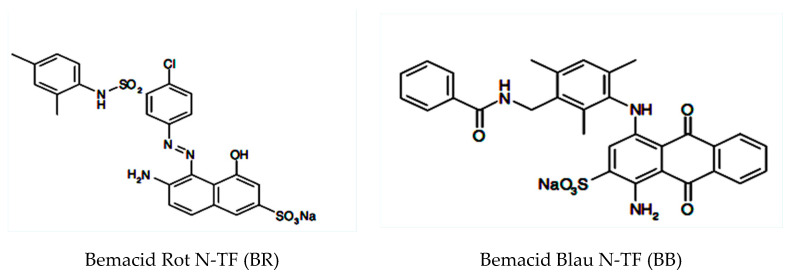
Chemical structures of the dyes.

The synthetic wastewaters were prepared by using bi-distilled water and dyes: Bemacid Rot N-TF (BR) with azo group (–N=N–) and auxochrome groups (–OH, –NH_2_, –SO_3_Na) and Bemacid Blau N-TF (BB) with auxochrome groups (–SO_3_Na, –NH_2_, –NH–) and anthraquinone chromophore group (Figure 1), from Bezema AG, Switzerland. BB and BR are used in dyeing of cotton, and they are highly resistant to biodegradation.

### 2.2. Materials Preparation Procedures

#### 2.2.1. Preparation of Pt-Cys NPs

Pt-Cys NPs were obtained using a microemulsion assisted photoreduction technique (MAPR), a procedure like one previously published by authors to synthesize AuNPs [30]. This consists of the reduction of platinum precursor under UVC light irradiation (λ = 254 nm) in water-in-oil (W/O) microemulsion template (82.5% n-heptane, 15% Brij 30, 2.5% HAuCl_4_ 1 g·L^−1^), in the presence of cysteamine monoclorhidrate ligand (Cys) used as stabilizer until the color of the microemulsion changes showing the formation of Pt-Cys NPs.

#### 2.2.2. Preparation of Photocatalytic Adsorbent Materials

The preparation method of the new composite material is as follows. First, fly ash particles, with size between 40 and 100 µm, and NaOH pellets were fused at high temperature, 500 °C, for 3 h, followed by cooling at room temperature. Then, the material was crushed and mixed with de-ionized water for 1 h until a suspension was formed. The enrichment of materials with TiO_2_ was done by adding, under stirring (300 rpm), Degussa P25 and storing in an autoclave, at atmospheric pressure and 100 °C, for 24 h. After this time, the stirring was interrupted, and the suspension was preserved for 24 h at 100 °C for nucleation in an alkaline environment. The suspension was filtered, rinsed several times with double distilled water and dried at 105–115 °C for 12 h.

To increase and extend the photocatalytic activity of the nanocomposite powder, by stopping the recombination of the e- with h+, positively charged platinum nanoparticles (Pt-Cys NPs) were added.

The resulting composite, with adsorbent and photocatalytic properties active in UV and Vis, is denoted by FADPt.

### 2.3. Structure and Surface Characterization

The crystalline structure and variations in chemical structure of FADPt composite were investigated by using an X-ray diffractometer (XRD Bruker D8 Discover Diffractometer, Kα1 = 1.5406 Å, 40 kW, 20 mA, step size 0.02, scan speed 2 sec/step, 2θ range ranging from 10 to 80°). The surface morphology (roughness and macro-pore size distribution) was studied using scanning electron microscopy (SEM S-3400N-Hitachi, accelerating voltage of 20 KV) and an atomic force microscope (AFM Ntegra Spectra, NT-MDT model BL222RNTE). The images were taken in semi-contact mode with Si-tips (NSG 10, at constant force 0.15 N/m with 10 nm tip radius). The size, shape and crystallinity of the samples were observed by high resolution transmission electron microscopy performed on a PHILIPS CM 120 ST HR-TEM. Energy dispersive X-ray (EDX, Thermo Scientific Ultra Dry) was used to outline the surface elemental composition. The XPS spectra were recorded on Thermo Scientific K-Alpha equipment, fully integrated with an aluminum anode monochromatic source. Survey scans (0–1200 eV) were performed to identify constitutive elements. Fourier-transform infrared spectroscopy was used to identify the new chemical changes. The FT-IR spectra of the new composite were recorded with a Spectrum BX Perkin Elmer BXII 75548, λ = 400–4000 nm. The surface energy was calculated. The reflectance spectrum was taken using a UV–Vis-NIR spectrometer (Perkin Elmer Lambda 25 UV/Vis), and the data were used to evaluate the optical band gap (Eg) of the samples.

Static contact angle measurements based on the sessile drop method were recorded and analyzed using an OCA-20 Contact Angle meter (Data Physics Instruments).

Microporosity and BET specific surface were measured using an Autosorb-IQ-MP, Quantachrome Instruments.

### 2.4. Tandem Adsorption and Photodegradation Experiments

**Figure 2:**
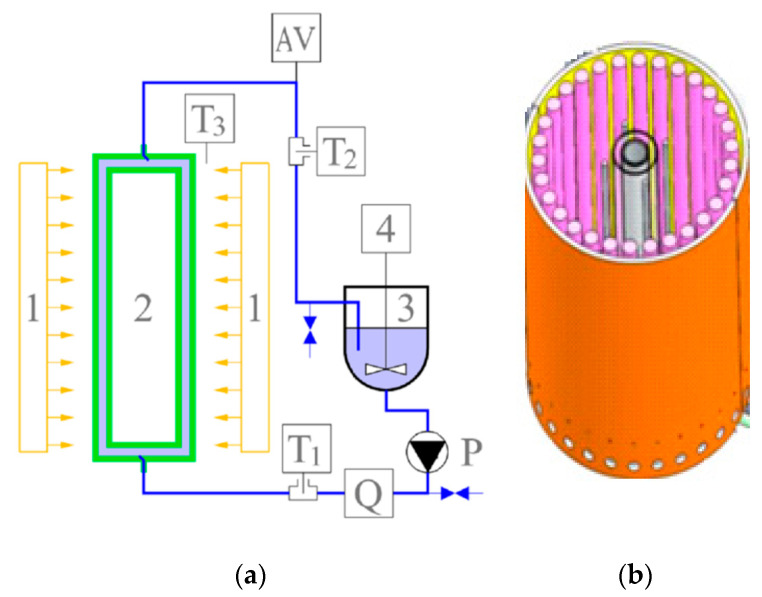
Functional scheme of the photo-reactor (**a**) and detail of the cylindrical quartz tube inside the lighting system (**b**). 1—LEDs and UV lamps; 2—Cylindrical quartz tube; 3—Storage tank; 4—Electric overhead stirrer; AV—Air vent; P—Hydraulic pump; Q—Flowmeter; T1—Inlet temperature sensor; T2—Outlet temperature sensor; T3—Ambient air temperature sensor [31].

The photodegradation experiments were run using a homemade vertical tubular photocatalytic reactor (Figure 2a) with two concentric quartz tubes working in continuous flow with suspensions. The radiation source consists of a series of 11 LEDs (LED-tube 600 mm 8 W/865 cool daylight tubes, Philips CorePro, emitting Vis light, 400–700 nm, λ_max_ = 5450 nm, G = 8 W/m^2^) and 11 UV tubes (F18W/T8/24/BL368—UVA lamp, BL368 tubes emit an upgraded highly concentrated radiation with a peak around 368 nm, G = 18 W), interchangeable, positioned around the external surface of the quartz tube (Figure 2b) to prevent scattering and to ensure uniform distribution. The radiance for each experiment was G = 208.7 W/m^2^ with energy consumption of 2.52 KWh. The substrate dose was 0.6 g/L.

The synthetic wastewater solution contained 50 mg/L BR or BB. Consequently, for each experiment, 3 g FADPt and 5 L solution were introduced into the reservoir of the reactor, without and with different volumes of H_2_O_2_ 30% (4, 8 and 12 mL), under continuous recirculation, assured by the peristaltic pump, with 61–65 L/h for 30 min in dark to reach the adsorption–desorption equilibrium before irradiation. Thus, the active species generated by photoirradiation could attack the organic pollutants if they are in the very close proximity of activated sites.

Independent adsorption experiments were conducted in continuous flow at room temperature, 23 ± 1 °C, without H_2_O_2_ to quantify the adsorption contribution to the photodegradation efficiency on FADPt. The composite dose and the initial concentrations of pollutants were like those in the photocatalysis process. Since the uptake rates of dye onto FADPt are of great importance with regard to potential scale-up and implementation of this system to remove pollutants from water, kinetic studies were conducted. Aliquots were taken at fixed moments (30 up to 360 min), a time long enough to reach adsorption of the dyes. After filtration on a Millipore Millex-GP, Hydrophilic PES filter (0.45 μm), the pollutant concentrations were measured and calculated on a calibration curve, at the maximum absorption peaks (for BR (λ_BR_ = 501 nm) and for BB (λ_BB_ = 629 nm)). The adsorption test was conducted using a quartz tube, and dye losses due to adsorption on the walls of the tube and on the filter paper were negligible. The tests were performed in triplicate, and the deviations were less than 3%. The removal efficiency of the dyes by adsorption/photodegradation was obtained in relation to Equation (1).

(1) $$\eta =\frac{C_{o}-C_{t}}{C_{o}}\times 100,\;\left[\%\right]$$

where *C_o_* is the initial concentration and *C_t_* is the dye’s concentration at moment t.

The adsorption and photodegradation processes were investigated at natural working pH of the suspension (pH = 7.89–8.04).

## 3. Results and Discussion

### 3.1. Characterization of the Composite

#### 3.1.1. Crystalline Structure

**Figure 3:**
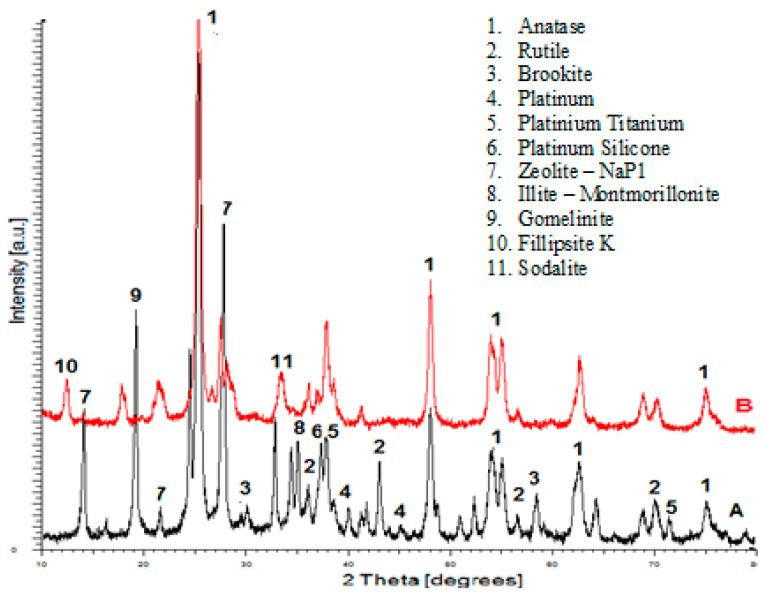
XRD patterns. (**A**) (black line): before adsorption/photocatalysis; (**B**) (red line): after adsorption/photocatalysis.

The diffraction peaks of the heterostructures (Figure 3A) confirm that in this system, the fusion above 550 °C, hydrothermal and ultrasound, was sufficient to obtain a polycrystalline surface, zeolites, TiO_2_, platinum-titanium (Pt-TiO_2_) and platinum-silicone (Pt-SiO_2_). In the diffraction pattern of the FADPt, two additional peaks corresponding to the crystallographic planes JCPDS 00-001-1194 of Pt at 2θ = 40.03° and 45.55° were detected and had a cubic crystallite cell with 19.59 nm. On the FADPt diffractogram, the shape of the peaks appears as overlapping, meaning that this substrate contains semiconductor oxides homogenously dispersed within the composite, which indicates the existence of a high amount of contact aluminosilicate (zeolite-NaP1, gomelinite, illite-montmorillonite) with TiO_2_. The alkaline hydrothermal treatment also initiated the TiO_2_ recrystallization process on the micro-sized aluminosilicates, extending the crystallites dimensions. Based on the XRD results, the crystallite sizes were calculated by using the Scherrer equation. The crystallite sizes are influenced by the ratio of the nucleation in alkaline hydrothermal process. It is also notable that FADPt contains all three TiO_2_ polymorphs: anatase has a tetragonal crystalline cell (JCPDS 00-001-0562) with 13.18 nm crystallite size, rutile has a tetragonal crystalline cell (JCPDS 00-001-1292) with 8.50 nm crystallite size and brookite has orthogonal crystalline cell (JCPDS 00-016-0617) with 8.15 nm crystallite size. The overall crystalline degree of FADPt is estimated at 79.33%, the rest being amorphous phases, and after adsorption/photocatalysis of BR the crystalline degree is 79.10% (Figure 3B).

The composition of the main components in the powder composite is presented in Table 1. Several studies have shown that the materials based on Pt or Au nanoparticles incorporated on composite with TiO_2_ have much improved charge transfer and thus increased catalyst performance [32].

#### 3.1.2. Surface Morphology

The porosity assays were performed using the adsorption–desorption technique on solid samples. The specific surface area (SBET) and pore distribution were analyzed, and the results are presented in Table 2. Corroborating the data provided by adsorption–desorption isotherms and pore volume, based on the BJH (Barrett-Joyner-Halenda) for the analyzed sample, it can be concluded that this is non-porous material with small microporosity areas.

The information on the polarity, surface energy and surface wetting behavior are important parameters for substrates used in adsorption/photocatalysis of the pollutants from aqueous solutions. The adsorption of the cations involves electrostatic forces; therefore, the surface energy of the composite (FADPt) can strongly influence the adsorption process. The contact angle values (*θ*) of the composite (FADPt) measured using water as wetting liquid were low (5–10°) proving a good surface wettability. The surface energy (*σ*) (polar and dispersive) was obtained from test liquids with different polarities: glycerol (with polar component of the surface $\sigma_{S}^{p}=41.50$ = 41.50 mN/m) and ethylene glycol (with polar component of the surface $\sigma_{S}^{p}=19.00$ mN/m). According to Owens and Wendt, the FADPt composite surface energy has a predominant polar nature, which indicates the hydrophilic surface and wettability, making them a suitable composite with dual applications (adsorbent and photocatalysts) in simultaneous removal of dyes from wastewater [33]. The polar component ($\sigma_{S}^{p}=41.50$ = 88.90 mN/m) of the surface energy with $\sigma_{S}^{p}\;>\sigma_{S}^{d}$ (Table 2) is the result of increased crystalline to 79.33% of the composite. The FADPt composite, with a large polar component and high crystallinity can better adsorb the dye molecules.

**Figure 4:**
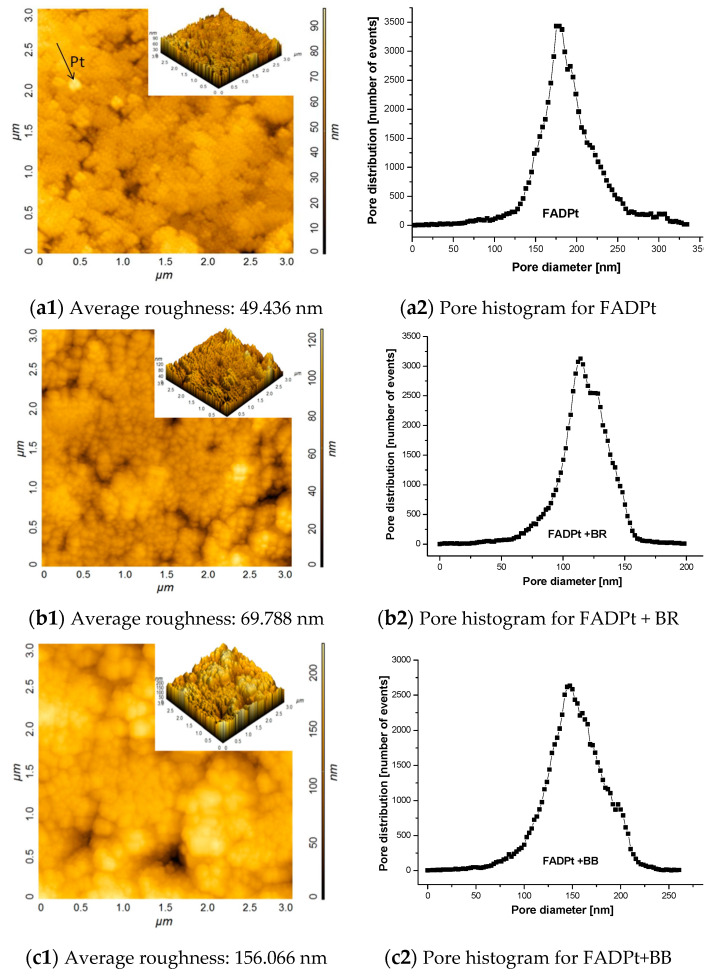
AFM topography, average roughness and pore distribution before and after adsorption/photocatalysis for: (**a**) FADPt; (**b**) FADPt loaded with BR; (**c**) FADPt loaded with BB.

The AFM images (Figure 4) show, in addition to uniform grains, that the composite also contains aggregates with various shapes and sizes (Table 3). These shapes can also be seen on the surface of the substrates loaded with adsorbent dyes.

The particle sizes after adsorption/photocatalysis increased (Table 3), and higher roughness was recorded from 49.436 (Figure 4a) to 69.788 (Figure 4b) and 156.01 nm (Figure 4c), respectively. Adsorption of the BB dye with a large molecule developed a surface with high roughness compared with adsorption of BR dye, the molecule with four aromatic rings and an azo group.

**Figure 5:**
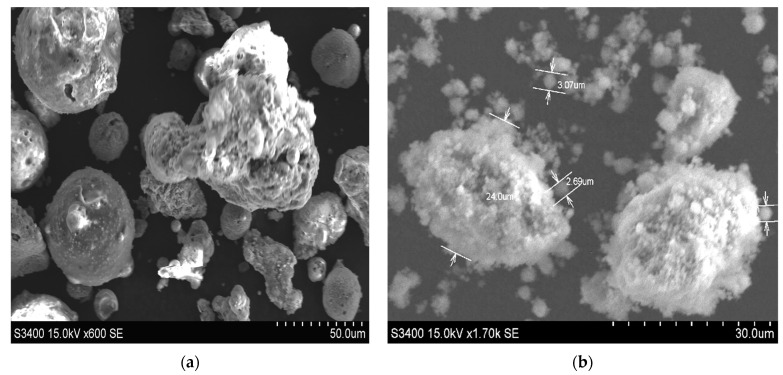
SEM images of (**a**) FAw; (**b**) FADPt.

The pore size distribution was estimated on AFM data using the method described by Otero et al. [34]. The results show that FADPt pores (Figure 4a) are narrower (<200 nm) than that of the washed fly ash (FA) 0.4–1.1 µm [27]. The incorporation of the quite uniform TiO_2_ Degussa P25 powder in the large pores of the FA can also be responsible for these changes, forming aggregation with anatase, rutile or brookite [35]. The size of individual grains is close to 2.69–3.07 µm, and that of the grains from agglomerates is above 24.00 µm (Figure 5b).

The AFM, SEM and EDX patterns show an ordered and homogeneous distribution of the TiO_2_ nanoparticles on the FADPt composite. The active sites suggest favorable adsorption and photodegradation processes of the dyes. This assumption is supported by other studies on the modification of the FA surface during the hydrothermal or even simple alkali treatment and/or the presence of other oxides (e.g., TiO_2_) [27]. Adsorption is likely to start inside the larger micropores, therefore the distribution curves are shifted to lower values of the diameters, with a predictable increase in the roughness (Figure 4c).

**Figure 6:**
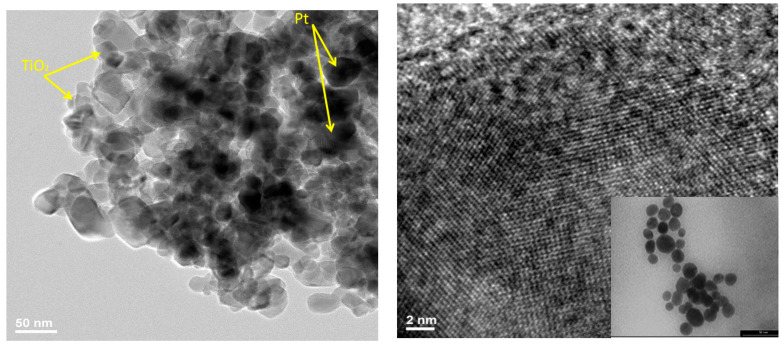
High magnification TEM image showing platinum nanoparticles aggregate on the TiO_2_.

The heterostructure of the composite was characterized by TEM and is shown in Figure 6. Pt nanoparticles are associated in mezoagregate with zeolite and TiO_2_. TEM images recorded for Pt-CS NPs show that they have a spherical shape and dimensions between 10 and 15 nm.

**Figure 7:**
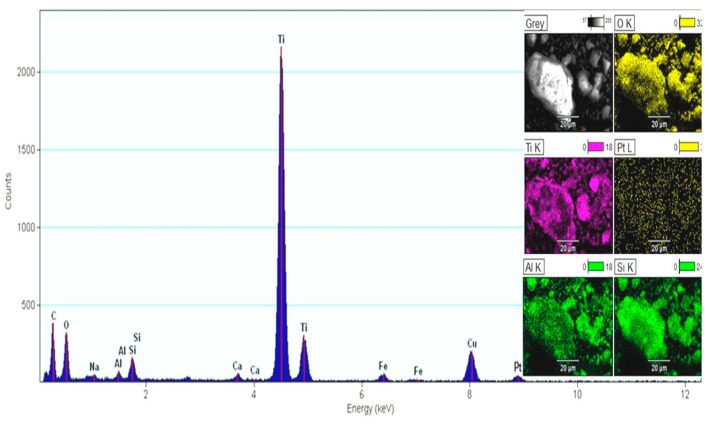
EDX spectrum and mapping images of FADPt.

HR-TEM images reveal {100} and {111}d-spacings, similarly reported by others for shaped Pt nanoparticles [36,37]. The images presented in Figure 6 show the characteristic sheets of the layered TiO_2_, zeolite, confirming that, although disordered, the solids maintain the intrinsic organization of the layered TiO_2_ and zeolite. The EDX spectrum and mapping images were created, and the results are included in Figure 7 and Table 4, respectively. The EDX mapping (Figure 7) suggests a homogeneous distribution of Pt nanoparticles located between the anatase, rutile and aluminosilicates.

The nitrogen and sulfur content data prove that the BR and BB dyes were adsorbed onto the substrate or were photodegraded. The organic or inorganic carbon on the surface of the composite increased after adsorption/photodegradation, suggesting accommodation or decomposition of the dyes in organic compounds with smaller molecules.

#### 3.1.3. UV–Vis Diffuse Reflectance Spectra

By coupling two, three semiconductors or semiconductors with noble metals with a suitable electronic band (SiO_2_, TiO_2_, WO_3_, Pt, Au, Ag), the photocatalytic activity under UV or Vis irradiation could be improved. The composite FADPt (Eg = 2.1 eV) is more active in visible light, and it can involve other compounds from the substrate at working pH = 7.89. More rigorously, the band gap of the semiconductor, in this case a mixture of semiconductors and Pt, was obtained from optical measurements based on the reflectance spectra. The results are presented in Table 2.

#### 3.1.4. X-ray Photoelectron Spectroscopy

**Figure 8:**
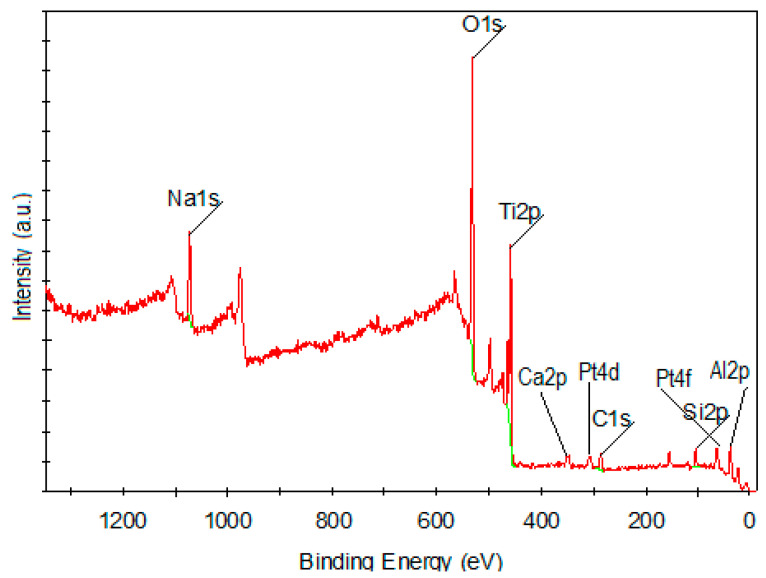
XPS spectrum of FADPt composite.

XPS was carried out, and the binding energies of the main peaks (Ti2p, O1s, Al2p, Si2p Pt4d and Pt4f) are listed in Table 5. The peak fixed at 459.1 eV corresponds to Ti^4+^ oxidation state in all three TiO_2_ polymorphs [24] and confirms the buildup of TiO_2_ nanoparticles on the zeolites (O1s, Al2p, Si2p). The O1s peak centered at 531 eV is assigned to oxygen from oxides with Si, Al, Na, Ca atoms or to oxygen from hydroxyl groups on the surface. The signal of Pt 4f is very small due to the low Pt NP content in the sample (Figure 8). The atomic percentages of the elements calculated by XPS were found to be smaller than EDX results (Figure 7).

#### 3.1.5. FT-IR Analysis

**Figure 9:**
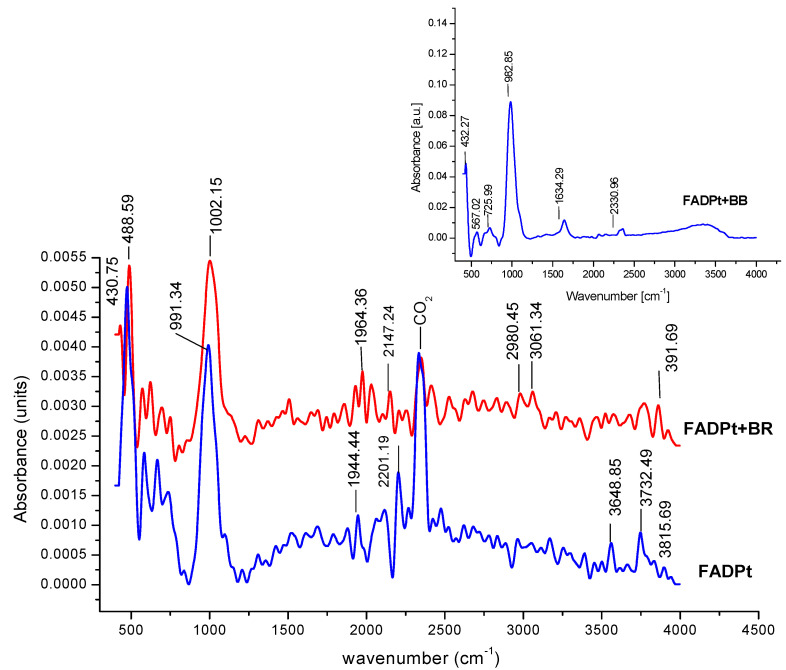
FTIR spectrum of FADPt, FADPt loaded with BR, FADPt loaded with BB.

The FT-IR spectra (Figure 9) of FADPt composite, before and after photodegradation/adsorption of dyes BB and BR, were analyzed to investigate the vibration frequency changes of the functional groups that identify the type of chemical bonds, indicating the complex nature of the adsorbent (Table 6).

The peaks located at 1615 and 1678 cm^−1^ are indexed for O–H (bonding water molecules). The sharp and intense peaks were recorded at 1002, 990, 982, 997 cm^−1^ and are attributed to the asymmetric stretch of Ti–O–Si; Si–Al–O; Al–O; Si–O [38,39,40,41]. The broad band around 425, 447 cm^−1^ is characteristic of O–Ti–O bridging vibration from rutile. The linear carbonyl Pt^3+^, Pt–CO, identified at 2176 cm^−1^ in FADPt, reveals a shift in the case of FADPt loaded with BR to a higher frequency (2246 cm^−1^), suggesting that the Pt nanoparticles interacted with BR dye. A weak band of Pt–(CO)–Pt was also registered at 1918 and 1873 cm^−1^ for the initial material and in FADPt loaded with BR, respectively.

### 3.2. Tandem Adsorption and Photodegradation on FADPt

#### 3.2.1. Dye Adsorption on FADPt

**Figure 10:**
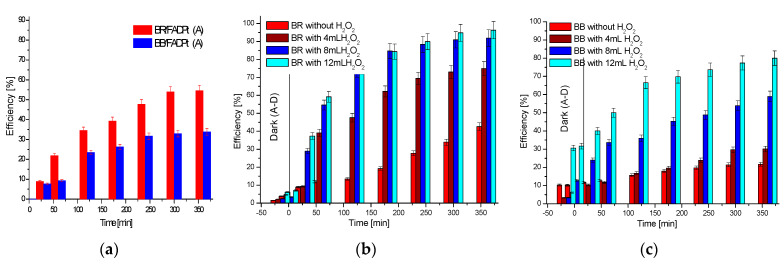
The efficiency of BB and BR dye removal: (**a**) by adsorption; (**b**,**c**) by photocatalysis.

The results of the adsorption process are presented for pH = 7.89 (Figure 10a). The experiments run under a radiance of 208.7 W/m^2^ (Figure 10b,c) showed an increased efficiency when H_2_O_2_ was added.

The adsorption efficiency (53.59% BR and 33.81% BB) depends on the composite characteristics (charge of the surface, morphology, porosity, distribution of the active sites) and the chromophore group of the dye. Both dyes have large molecules and different chromophore groups, anthraquinone in BB dye and azo in BR, groups that have different behavior in contact with active sites on the substrate. During the adsorption process, the dye could diffuse onto adsorbent, in micropores and/or could develop weak physical bonds between functional groups (-NH_2_, -NH-, -OH, -SO_3_Na) and the active sites disposed on TiO_2_ and FADPt aggregates [40].

#### 3.2.2. The Tandem Adsorption and Photocatalysis Processes

Based on these studies, the optimum volume of the H_2_O_2_ 30%, which required the addition of 5 L BR solution with 3 g substrate followed by 360 min of irradiation, is 8 mL (Table 7). The percentage of 91.88%, an improved efficiency by 48.88% compared to photocatalysis, was recorded for BR removal, thus confirming the positive effect of H_2_O_2_ and reactivity of the BR molecule in photodegradation process. On the other hand, adding 12 mL H_2_O_2_ at 5 L BB solution, in the same working conditions, led to an increase in the efficiency, by 54.90%, for BB dye removal. The molecule of BB is more complex, and the maximum efficiency in this case is 79.99%. A percentage of approximately 5% contribution of adsorption in darkness for 30 min was estimated for BR dye, while for BB it was between 10 and 30%, depending on H_2_O_2_ concentration.

The composite, containing anatase (43.99%), rutile and brookite, is activated by irradiation with an energy higher than the band gap value Eg = 2.1 eV, corresponding to λ = 399.9 nm. Under irradiation, the electron-hole pairs are photogenerated on the anatase surface (Equation (2)). The oxidative holes (h+)_VB_ can react with H_2_O to form active species OH∙, react with hydroxide ions on the surface (Si–OH) or can directly react with the dye molecules producing degradation product (Equations (3)–(5)) [42]. The dye molecules (R–H) are degraded by the hydroxyl radicals to organic intermediates, smaller organic molecules or inorganic compounds, such as CO_2_ and H_2_O or to others (Equation (6)). The organic intermediates can be more discolored with one aromatic ring and are sometimes very difficult to degrade [40,43].

2TiO_2_ + hν → TiO_2_(h+)_VB_ + TiO_2_(e-)_CB_ (2)

TiO_2_(h+)_VB_ + H_2_O_(ads)_ → HO∙ + H+ + TiO_2_ (3)

TiO_2_(h+)_VB_ + HO^−^ → HO∙+ TiO_2_ (4)

TiO_2_(h+)_VB_ + R–H → Oxidation product (5)

R–H + HO → Degradation product(6)

**Figure 11:**
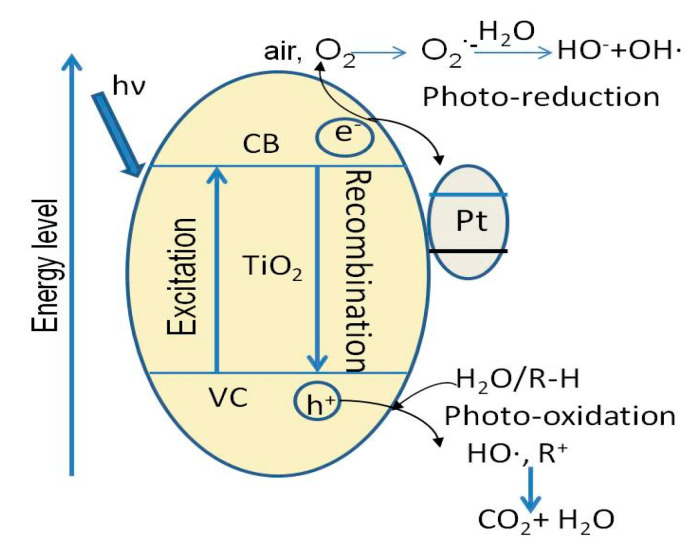
Schematic diagram of photocatalytic mechanism.

The high charge recombination rate and the wide band gap (rutile 3.0 eV and anatase 3.2 eV) impose a fundamental restriction on the overall photocatalytic efficiency of TiO_2_. By combining TiO_2_ with noble metal nanoparticles, such as Pt [23,44], Au [24], Ag [18] and Pd [25], Schottky barriers [45] could be formed, which drive photogenerated electrons from TiO_2_(e-)_CB_ to the noble metal (Pt), decreasing the recombination speed of e- with h+, and thus an enhanced photocatalytic activity is obtained (Equation (7), Figure 11).

Pt^4+^ + TiO_2_(e^−^)_CB_ → Pt^3+^ + TiO_2_ (7)

#### 3.2.3. The Effect of Concentration of the H_2_O_2_ on the Photocatalytic Process

Photocatalysis is enhanced by a larger number of hydroxyl radicals because they are very powerful when oxidized, with E_ox_ = 2.8 V. One way to increase their rate is addition of hydrogen peroxide under UV radiation (Equation (8)):

H_2_O_2_ + hν → 2HO (8)

The optimal parameters for degradation of textile dyes (anionic dyes) are concentration of dye, H_2_O_2_ concentrations and working pH. According to some authors, the optimal pH is between 3 and 9 [46,47]. A lot of researchers have investigated the effect of H_2_O_2_ dosage on the decolorization of dyes [46]. The degradation efficiency of the dyes increases with addition of H_2_O_2_ to a certain value, due to the increase in the hydroxyl radical concentration (Equation (8)). The excess of H_2_O_2_ probably promotes the reaction of hydroxyl radicals with H_2_O_2_ molecules leading to formation of hydroperoxyl radicals (Equation (9)); these can also react with HO∙, reducing the amount of HO and thus decreasing the efficiency of dye degradation (Equation (10)).

HO∙+ H_2_O_2_ → H_2_O + HO_2_ (9)

HO_2_·+ HO∙ → H_2_O + O_2_ (10)

The photodegradation process of dyes with H_2_O_2_ also depends on their structure (Table 7), but most important is the working pH. In alkaline conditions, the hydrogen peroxide can decompose to water and oxygen, and the production of hydroxyl radicals is decreased [46] (Equation (11)).

H_2_O_2_ → H_2_O + O_2_ (11)

The optimum working pH is close to neutral, in this case pH = 7.89.

In the same conditions of irradiation, the BB dye is less degraded in all experiments. The degradation mechanisms are very different; the azo group, with labile π-bonds, is the first target for ∙HO∙ in the case of BR (azo-dye). The sulfonic groups also react to further produce phenol or other aromatic compounds [42,43], and the amino groups eventually form ammonia [44]. In BB dye, the HO∙ radicals attack the -NH- positions and sulfonic groups leading to a broad range of intermediates derived from the anthraquinone nuclei [40,47,48].

### 3.3. Kinetics Modeling of the Adsorption Processes

The kinetics models are fitted to the experimental data by the nonlinear regression analysis, according to the pseudo-second-order kinetics, given by Equation (12) [49]:

(12) $${\frac{t}{q}}_{t}=\frac{1}{k_{2}q_{e}^{2}}+{\frac{t}{q}}_{e}$$

where *k*_2_ is the rate constant for the pseudo-second-order adsorption (g·mg^−1^min^−1^) and *q_t_* and *q_e_* are the quantity of adsorbed dyes (mg∙g^−1^) at time *t* (min) and equilibrium.

The linearization proved that the pseudo-second kinetic order describes well the adsorption mechanism for both dyes (Table 8). These results outline again the importance of adsorption as a preliminary step in photocatalysis.

### 3.4. Kinetics Modeling of the Photocatalysis Processes

The kinetics data were modelled using a first kinetics derived from the Langmuir–Hinshelwood model (Equation (13)) [50]:

ln C/Co = −k_obs_ t (13)

where Co and C are initial dye concentration and the concentration at the moment t and k_obs_ is the overall observed pseudo-first-order degradation rate constant.

**Figure 12:**
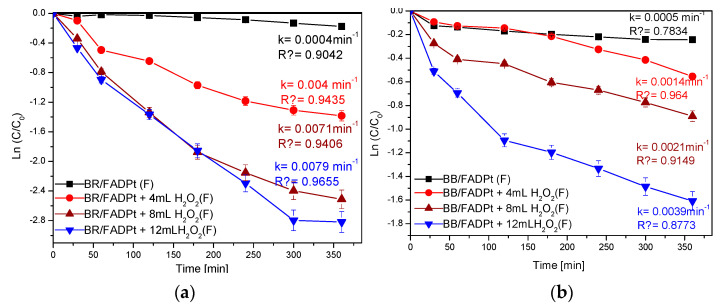
Photocatalysis of dyes (**a**) BR; (**b**) BB (R^2^ is the squared value of the linear regression).

The photodegradation results of the dyes, in working conditions, without and with oxygen peroxide (Figure 12), show that the mechanism follows the pseudo-first-order model.

The BR photodegradation also proved to be faster (k = 0.0079 min^−1^) as compared to BB (k = 0.0039 min^−1^), thus allowing a more significant quantity of oxidizing species to be involved. In the working conditions, pH = 7.89, the oxygen production from H_2_O_2_ is employed, which afterwards can contribute to decreasing recombination. H_2_O_2_ was used as an electron scavenger to accelerate decolorization and degradation of the BR and BB dyes.

## 4. Conclusions

Fly ash wastes were efficiently recycled to obtain an effective photocatalytic composite by adding nanosized Degussa P25 and positively charged platinum nanoparticles (Pt-Cys NPs). The mild hydrothermal process can be used to obtain a material with specific surface 10 times larger than the initial fly ash.

The FADPt material was tested in tandem adsorption and photocatalysis experiments for removing two industrial dyes, Bemacid Red (azo-dye) and Bemacid Blau (antraquinone-dye). The pseudo-second-order kinetic model governs the adsorption process of removing pollutants from complex systems. The rate constant of the pseudo-first-order process increased from 0.0004 min^−1^, when the degradation process developed without H_2_O_2_, to 0.0079 min^−1^, with H_2_O_2_.

Comparative investigations using similar materials show that FADPt substrate can be considered suitable for tandem adsorption and photocatalysis process, the dye removal efficiency being significantly improved by H_2_O_2_ addition. The addition of H_2_O_2_ in the working conditions improves the process efficiency up to 96.04% after 360 min. The PtNP contribution is revealed by the band gap energy change to Vis light (400 nm), thus suggesting the possibility of photocatalysis under the action of a fraction of natural light.

## Figures and Tables

**Table 1:** The composition of the FADPt.

Components of FADPt	Anatase	Rutile	Brookite	Pt (NPs)	Zeolite NaP1	Other
Composition (%)	43.99	1.81	1.79	0.88	17.14	35.27

**Table 2:** Surface characteristics of FA and FADPt.

Sample	S_BET_ (m^2^/g)	V_Micropores_ (cm^3^/g)	D_Average pores_ (nm)	Surface Energy		E_g_ (eV)
				Polar (mN/m)	Dispersive (mN/m)	
FA	6.14	0.0042	27.2	58.43	5.54	2.45
FADPt	40	0.17	35	88.90	1.42	2.12

**Table 3:** The parameters of grains analyzed based on AFM images.

Sample	Area (µm^2^)	Volume (µm^2^·nm)
FADPt/grain	0.049	3.269
FADPt loaded with BR/grain	0.872	6.946
FADPt loaded with BB/grain	0.916	18.621

**Table 4:** Surface composition, in element Wt.% and atomic %, of the FADPt surface before and after loading with BR and BB dyes.

Element Line	Element Wt. before (%)	Atom before (%)	Element Wt. (%) Substrate Loaded with BR	Atom (%) Substrate Loaded with BR	Element Wt. (%) Substrate Loaded with BB	Atom (%) Substrate Loaded with BB
C K	1.94	4.84	60.44	62.90	2.15	5.61
N K	0	0	18.18	17.54	11.38	16.39
O K	18.89	35.38	14.2	12.1	47.07	58.38
Na K	3.01	3.92	0.35	0.23	2.42	2.12
Mg K	0.99	1.32	0.08	0.05	0.11	0.09
Al K	2.61	2.9	0.77	0.38	4.18	2.13
Si K	12.81	13.62	0.85	4.41	4.92	3.54
S K	0	0	0.01	0.3	0.11	0.07
Ca K	22.9	17.13	0.32	0.41	1.58	0.8
Ti K	32.36	20.2	4.75	1.47	25.98	10.86
Pt M	4.49	0.69	0.05	0.21	0.1	0.01
Total	100.00	100.00	100.00	100.00	100.00	100.00

**Table 5:** Binding energy and the atomic % of FADPt.

Element	Binding Energy (eV)	Atomic %
O1s	531	52.07
Ti2p	459.1	13.24
Na1s	1072.77	9.79
Si2p	103.18	8.58
C1s	286.03	8.64
Ca2p	347.91	1.81
Al2p	75.11	4.01
Pt4d	315.08	1.45
Pt4f	75.08	0.40

**Table 6:** Characteristics of IR bands associated with FADPt, FADPt with loaded BB and FADPt with loaded BR.

Characteristics Groups	FADPt (cm^−1^)	FADPt + BB (cm^−1^)	FADPt + BR (cm^−1^)
Si–OH groups	3815	-	3931
Si–(OH)Al hydroxyl group stretching	3730	-	3755
OH groups bridging hydroxyls in zeolite cages to the same Al–OH–Si	3648	-	3661; 3509
Linear carbonyl Pt^4+^, Pt–CO, [Pt_3_CO)_6_]^2−^ Pt/Al_2_O_3_—(2093 cm^−1^); CO adsorbed on Pt atoms Pt^2+^–CO: 2155, 2141 cm^−1^	2176 2195	-	2246; 2184; 2111
	2093 2069 2155	-	2054
Weak bands Pt–(CO)–Pt	1918		1873
Water molecules	1615	1642	1678
Si–Al–O; Al–O asymmetric stretch Ti–O–Si	990 1002	982 992	997 971
O–Ti–O from rutile	425	-	447
Ti–O–Ti bridging vibration	811	730	788,740
Si–O bond of the zeolite structure	689	662	684
HO–Pt–OH stretch vibration	577	561	582

**Table 7:** The influence of oxygen peroxide volume on dye degradation.

	BR—Removal (%)					BB—Removal (%)				
	V_H2O2 (30%)_ (mL)					V_H2O2 (30%)_ (mL)				
Time (min)	4	8	12	8 − 4	12 − 8	4	8	12	8 − 4	12 − 8
120	47.59	73.76	74.50	26.16	0.74	16.80	36.01	66.54	19.21	30.53
180	62.14	84.65	84.33	22.51	−0.32	19.63	45.32	69.77	25.69	24.44
240	69.43	88.39	89.92	18.96	1.53	23.94	48.80	73.65	24.85	24.85
300	72.98	90.89	93.89	17.91	3.00	29.74	53.87	77.37	24.13	23.49
360	74.96	91.88	94.04	16.92	2.15	30.16	58.94	79.99	28.77	21.05

**Table 8:** Kinetic parameters of the dye removal in adsorption processes.

Pollutant (Dye)	Pseudo-Second-Order		
	k_2_ (g·mg^−1^·min^−1^)	q_e_ (mg/g)	R^2^
BR (A)	2.532	8.024	0.849
BB (A)	4.385	5.204	0.929
